# Renal and Splenic Infarctions: Unmasking Rheumatic Mitral Valve Disease in a Young Adult

**DOI:** 10.7759/cureus.107177

**Published:** 2026-04-16

**Authors:** Esperance M Madera, Elda Mullaj, Anish Munagala, Priyanka Bhargat, Joseph Trifari

**Affiliations:** 1 Internal Medicine, Mount Sinai Hospital, Chicago, USA; 2 Cardiology, Mount Sinai Hospital, Chicago, USA; 3 Internal Medicine, Detroit Medical Center, Wayne State University School of Medicine, Michigan, USA; 4 Internal Medicine, St. George’s University School of Medicine, True Blue, GRD

**Keywords:** atrial fibrillation, mitral regurgitation (mr), renal cortical infarct, rheumatic valvular heart disease, splenic infarct

## Abstract

Systemic thromboembolism is an uncommon but clinically significant complication of rheumatic heart disease (RHD), most often seen in association with infective endocarditis. We describe a 39-year-old woman with no known cardiovascular comorbidities who presented with acute left-sided abdominal pain. Imaging revealed concurrent renal and splenic infarctions. Further evaluation uncovered paroxysmal atrial fibrillation, severe mitral regurgitation, and moderate to severe mitral stenosis with marked left atrial enlargement. Transthoracic and transesophageal echocardiography confirmed the diagnosis. She was managed with anticoagulation, rate control, and subsequently underwent surgical intervention consisting of mitral valve replacement, tricuspid annuloplasty, and left atrial appendage closure. Postoperatively, the patient had an uneventful recovery, with stable vital signs, no evidence of thromboembolic events, and sustained sinus rhythm at discharge. Repeat echocardiography showed a well-seated and normally functioning mitral bioprosthesis, with no rocking motion and no significant trans-prosthetic or peri-prosthetic regurgitation. This case highlights the importance of considering RHD as a potential cause of systemic embolism, even in younger patients without traditional cardiovascular risk factors. It emphasizes the need for a thorough cardiovascular evaluation in patients presenting with systemic embolism to ensure early diagnosis and appropriate management.

## Introduction

Rheumatic heart disease (RHD) is a chronic sequela of acute rheumatic fever (ARF), which results from an autoimmune response to untreated or inadequately managed group A streptococcal pharyngitis. ARF is characterized by systemic inflammation affecting the joints, skin, central nervous system, and heart. Cardiac involvement, particularly valvulitis, is the most important determinant of long-term outcomes, as it can progress to permanent structural damage of the valves [1].

While RHD remains a significant health concern worldwide, its epidemiology varies significantly between high-income and low- and middle-income countries (LMICs). In high-income countries, RHD has become relatively rare due to improved living conditions, early diagnosis, and widespread use of prophylactic antibiotics for streptococcal throat infections. In contrast, it remains a leading cause of acquired heart disease in children and young adults in LMICs, where the incidence of ARF is higher and access to healthcare services, including preventive care, is limited. It is responsible for an estimated 250000 deaths annually, with the vast majority of these occurring in LMICs [2].

Although many patients with ARF recover without lasting effects, recurrent or severe episodes predispose to progressive valvular dysfunction, most commonly involving the mitral valve. RHD may remain clinically silent for years and present only in adulthood, once complications such as atrial fibrillation, heart failure, or systemic embolization occur [2,3]. In patients presenting with systemic thromboembolism, a thorough cardiac workup is essential to identify potential cardioembolic sources, including underlying valvular heart disease such as RHD. While echocardiographic screening programs have improved early detection, systemic embolization remains an uncommon initial manifestation of previously unrecognized RHD. We describe a case in which renal and splenic infarctions were the sentinel events that prompted cardiac evaluation and ultimately led to the diagnosis of severe rheumatic mitral valve disease.

## Case presentation

**Figure 1:**
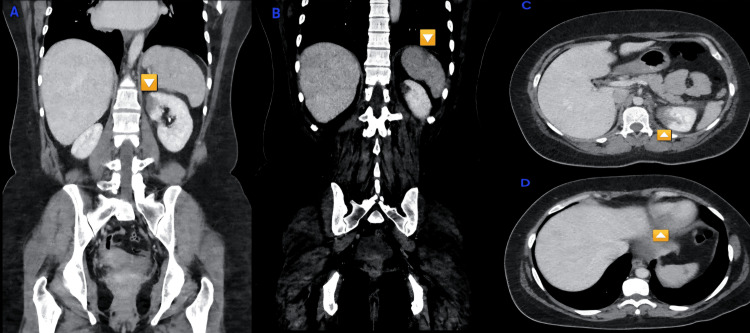
Multimodality imaging findings (A, B) Contrast-enhanced CT demonstrating a left renal infarct. (C) Contrast-enhanced CT showing a splenic infarct. (D) CT angiography highlighting a splenic perfusion defect consistent with infarction. CT: computed tomography

A 39-year-old woman with no significant past medical history presented to the emergency department with acute, non-traumatic left-sided abdominal pain associated with nausea. On initial examination, she was afebrile with a blood pressure of 129/83 mmHg, pulse of 85 beats per minute, respiratory rate of 16 breaths per minute, and oxygen saturation of 99% on room air. Her pulse was regular at presentation. Physical examination revealed abdominal tenderness in the epigastric region and left lower quadrant. Cardiac auscultation demonstrated normal heart sounds in all precordial areas. A urine pregnancy test and urinalysis were negative. Additional laboratory workup showed a PT of 10.6 seconds, PTT of 35.8 seconds, INR of 1.3, D-dimer of 2.12, lactic acid of 0.8, and lipase of 8; the remainder of the initial laboratory studies was unremarkable. Computed tomography (CT) of the abdomen demonstrated a renal infarct (Figure 1A, 1B) and a splenic infarct (Figure 1C, 1D).

**Figure 2:**
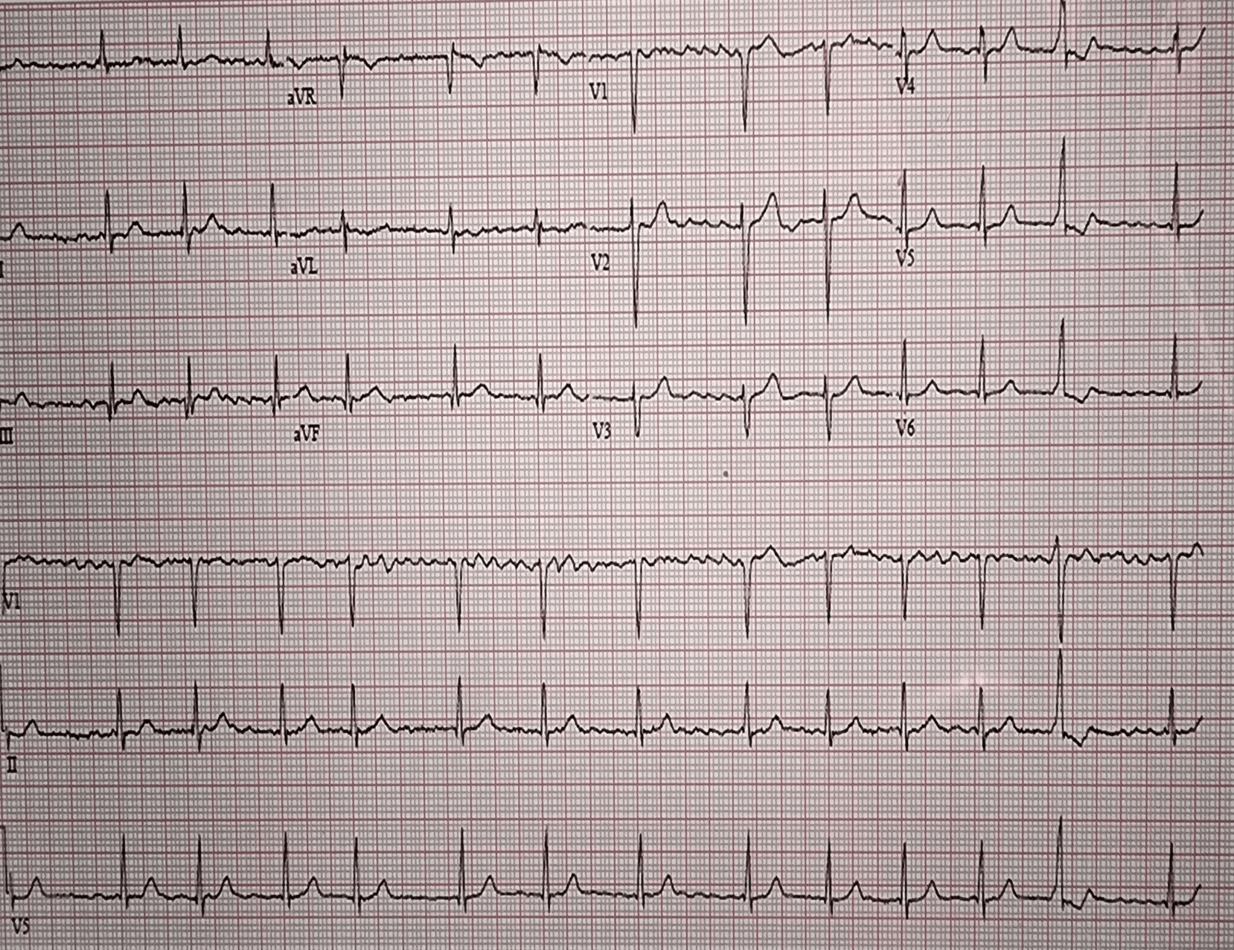
ECG demonstrating atrial fibrillation Electrocardiography showed a ventricular rate of 67 bpm, atrial rate of 227 bpm, and QTc of 374 ms. ECG: electrocardiogram

Given concern for a cardioembolic source, further cardiac evaluation was performed. Subsequent electrocardiography revealed atrial fibrillation (Figure 2).

**Figure 3:**
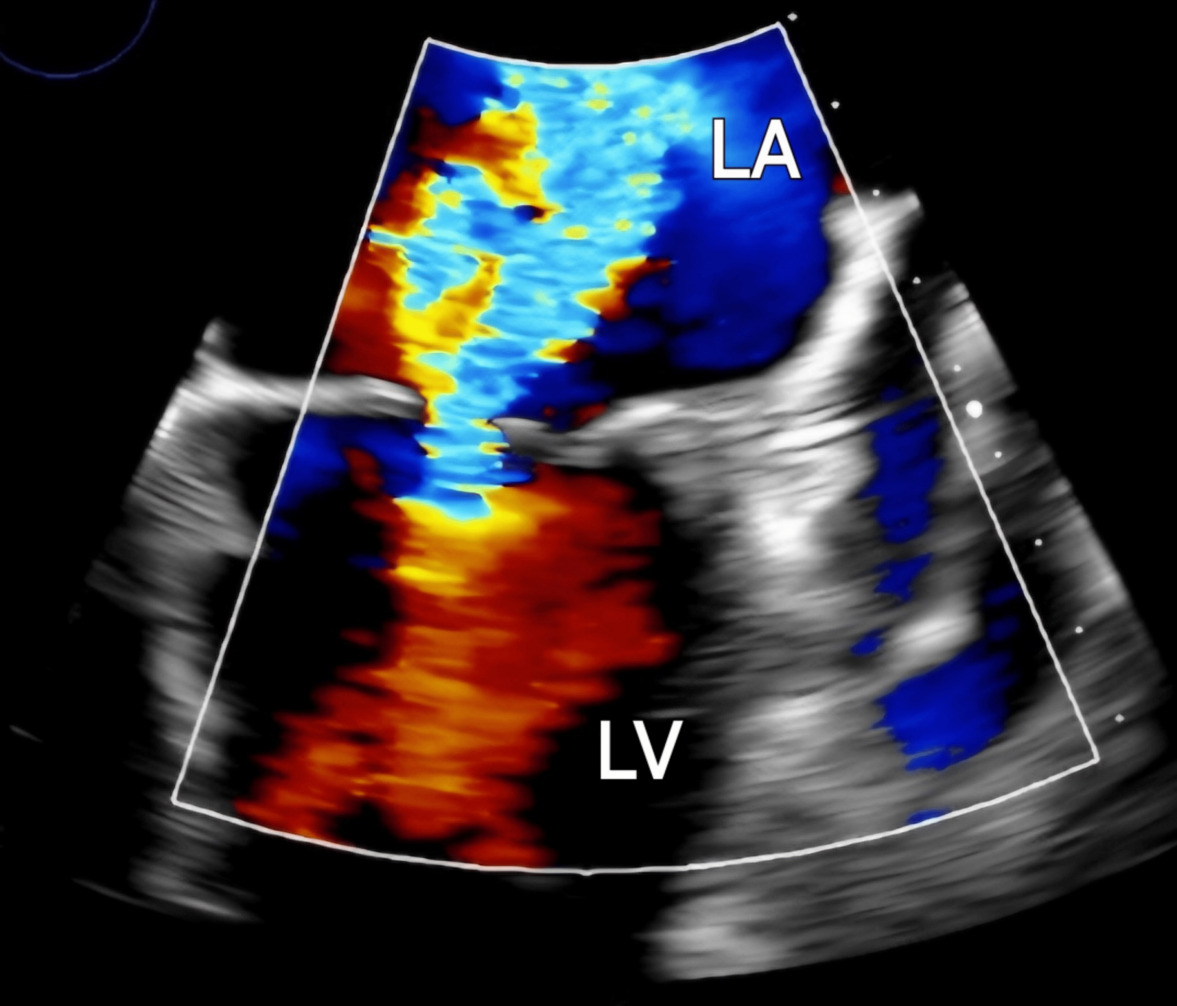
Transthoracic echocardiogram revealing severe mitral regurgitation and moderate to severe mitral stenosis Thickened and calcified mitral leaflets with diastolic doming (hockey-stick deformity) and restriction of the posterior leaflet, with findings consistent with rheumatic heart disease. In addition, a central leaflet malcoaptation gap is present, resulting in significant regurgitation. Doppler evaluation demonstrates severe regurgitation with multiple centrally directed jets. The PISA radius is 1.28 cm, and the peak mitral regurgitation velocity is 454 cm/s. The effective regurgitant orifice area is 0.75 cm², and the regurgitant volume is 83 mL. PISA: proximal isovelocity surface area; LV: left ventricle; LA: left atrium

Transthoracic echocardiogram showed a massively dilated left atrium with moderate to severe mitral stenosis, severe mitral regurgitation, and pulmonary hypertension (Figure 3).

Subsequently, transesophageal echocardiography demonstrated rheumatic mitral valve disease with mixed pathology, predominantly severe mitral regurgitation. The renal and splenic infarctions were determined to be the result of thromboembolism arising from atrial fibrillation secondary to rheumatic heart disease. Medical management with metoprolol and anticoagulation with therapeutic Lovenox was started. The patient underwent bioprosthetic mitral valve replacement, tricuspid valve annuloplasty, left atrial appendage clipping, and partial maze procedure, with restoration of sinus rhythm. A bioprosthetic mitral valve was selected after individualized multidisciplinary discussion, balancing the greater durability of a mechanical prosthesis against the risks and long-term burden of lifelong anticoagulation. Anticoagulation management was subsequently individualized by the treating team during follow-up; however, we acknowledge that current evidence does not support routine immediate discontinuation of anticoagulation solely on the basis of partial maze and left atrial appendage closure.

## Discussion

Renal infarction is an uncommon diagnosis in the emergency setting, but it should be considered in patients presenting with unexplained abdominal or flank pain. Its causes include trauma, vascular injury, underlying renal pathology, and thromboembolic events related to atrial fibrillation, infective endocarditis, or hypercoagulable states [4,5]. Although rare, retrospective studies have identified atrial fibrillation as one of the most common etiologies of renal infarction [6].

Splenic infarction is similarly uncommon, with an estimated incidence of 0.004%-0.01% among hospitalized patients [7]. Its presentation is often nonspecific, which may delay diagnosis. Autopsy studies suggest that splenic infarction contributes to substantial morbidity and mortality but is frequently unrecognized during life [5]. More recent retrospective analyses have also identified atrial fibrillation as a leading predisposing factor, along with hematologic and vascular disorders [8]. In some patients, splenic infarction may be the first manifestation of previously undiagnosed systemic or cardiac disease [6].

In the present case, the concurrent occurrence of renal and splenic infarctions served as the initial clue to an underlying cardioembolic source. Subsequent evaluation demonstrated rheumatic mitral valve disease with mixed pathology, including both mitral stenosis and severe mitral regurgitation, complicated by atrial fibrillation. This distinction is important, as rheumatic valve disease does not always present as isolated mitral stenosis. Mixed mitral valve disease can also create the substrate for left atrial enlargement, atrial fibrillation, and systemic thromboembolism.

Atrial fibrillation is a well-recognized complication of rheumatic mitral valve disease and contributes significantly to morbidity through thromboembolic risk and adverse hemodynamic effects [9]. In patients with mixed mitral valve disease, management must account for the combined effects of stenotic and regurgitant lesions rather than focusing solely on isolated mitral stenosis. Treatment is therefore individualized according to symptom burden, valve anatomy, severity of each lesion, presence of pulmonary hypertension, rhythm status, and overall surgical candidacy. Medical therapy may include rate or rhythm control, along with anticoagulation to reduce embolic risk, while definitive management often requires surgical intervention when valvular dysfunction is advanced or when anatomy is unsuitable for percutaneous approaches. In our patient, the extent of rheumatic valvular disease and associated complications prompted surgical management with mitral valve replacement, tricuspid annuloplasty, left atrial appendage clipping, and partial maze procedure, with a favorable postoperative outcome. Current guidelines also note that bioprosthetic mitral valve replacement is generally followed by three to six months of vitamin K antagonist therapy, underscoring the importance of careful postoperative anticoagulation planning in addition to rhythm management [10].

This case highlights the importance of maintaining a broad differential diagnosis in younger patients presenting with systemic embolic events in the absence of traditional atherosclerotic risk factors. It also underscores the need for comprehensive cardiac evaluation in patients with renal and splenic infarctions, as these events may represent the first manifestation of previously undiagnosed rheumatic heart disease. Early recognition of mixed rheumatic mitral valve disease and its embolic complications is essential to enable timely treatment and prevent further morbidity.

## Conclusions

The key teaching point from this case is that renal and splenic infarctions in a young patient without clear vascular risk factors should prompt evaluation for an underlying cardioembolic source, including occult rheumatic heart disease involving the mitral valve. Early use of an ECG and echocardiography in this setting may uncover clinically silent but advanced valvular pathology, allowing timely treatment and reducing the risk of further thromboembolic events.
